# Treatment of Peri-Implant Mucositis with Standard of Care and Bioptron Hyperlight Therapy: A Randomized Clinical Trial

**DOI:** 10.3390/ijerph19095682

**Published:** 2022-05-07

**Authors:** Gianna Maria Nardi, Marta Mazur, Giulio Papa, Massimo Petruzzi, Felice Roberto Grassi, Roberta Grassi

**Affiliations:** 1 Department of Oral and Maxillofacial Sciences, Sapienza University of Rome, 00161 Rome, Italy; profnardi.giannamaria@gmail.com (G.M.N.); marta.mazur@uniroma1.it (M.M.); gpapa82@gmail.com (G.P.); 2 Interdisciplinary Department of Medicine, Aldo Moro University of Bari, 70121 Bari, Italy; massimo.petruzzi@uniba.it; 3 Department of Basic Medical Sciences, Neurosciences and Sense Organs, Aldo Moro University of Bari, 70122 Bari, Italy; feliceroberto.grassi@gmail.com; 4 Department of Biomedical Sciences, University of Sassari, 07100 Sassari, Italy

**Keywords:** peri-implant mucositis, dental implants, clinical trial, Bioptron, oxidative stress, photobiomodulation, SAT, non-surgical periodontal therapy, dental hygiene, salivary test

## Abstract

The aim of this study was to evaluate in a cohort of patients with peri-implant mucositis: (a) the efficacy of professional mechanical debridement therapy assisted using Bioptron Hyperlight Therapy on the reduction in periodontal indexes and (b) the reduction in total oxidative salivary stress. Forty subjects with a diagnosis of peri-implant mucositis were enrolled and randomly assigned to the Study Group (mechanical debridement therapy assisted using Bioptron Hyperlight Therapy) or Control Group (mechanical debridement therapy alone). The study duration was 6 months. Data on plaque index (PI), bleeding on probing (BoP), probing pocket depth (PPD), and pain relief on Visual Analogue Scale (VAS) were recorded at T_0_, T_1_ (14 days), T_2_ (1 month), and T_3_ (6 months). Group differences were assessed using Student’s *t*-test and Pearson’s Chi-squared test of homogeneity. PI and PPD decreased in the Study Group at the [T_0_; T_1_] time interval and during the overall time of observation significantly more than in the Control Group; BoP and pain on VAS decreased significantly faster in the Study Group than in the Control Group. Differences in Salivary Antioxidant Test (SAT) changes were not significant at any time interval. Patients’ gender and smoking habit were not correlated with the clinical outcomes. Clinical parameters related to peri-implant mucositis significantly improved in the Study Group, which demonstrated the clinical efficacy of the Bioptron Hyperlight Therapy as an adjunct to standard of care for the treatment of peri-implant mucositis. The RCT was registered at the US National Institutes of Health #NCT05307445.

## 1. Introduction

Peri-implant mucositis is defined as a biofilm-induced inflammatory lesion around dental implants. Treatment of peri-implant mucositis must be based on a precise clinical framework and a diagnostic process that clearly identifies the clinical state and distinguishes it from peri-implant health as well as from peri-implantitis state [1].

The American Academy of Periodontology describe peri-implant mucositis as an inflammation of the soft tissues around dental implants, with no further bone resorption apart from physiological bone resorption resulting from implant placement [2]. The aetiology of peri-implant mucositis is the accumulation of a bacterial biofilm around the implant [3].

Peri-implant mucositis diagnosis should be based on clinical inflammatory signs. The clinical picture is determined by localized swelling, redness, and shininess of the soft tissue surface, combined with any bleeding on probing and an increase in probing depths compared to baseline and suppuration. Intra-oral radiographic evaluation should not demonstrate evidence of bone loss beyond crestal bone level changes resulting from the physiological remodelling process after implant placement. The patient may report soreness [4].

Identified risk factors are biofilm accumulation, poor maintenance, smoking, and radiation [3], while further evidence is required for diabetes, history of periodontitis, lack of keratinized mucosa, presence of excess luting cement and shape of the implant superstructure [3].

The therapy consists of both professional intervention and at home oral-hygiene techniques with the adjunctive use of antimicrobials [5].

Professional interventions are based on mechanical debridement of the supra and subgingival implant surface, the implant neck and the abutment, without altering the implant surface. The debridement systems include curettes and ultrasonic devices, combined with the use of polishing tips for the implant surface and/or the prosthetic components [5]. Antimicrobials are used in combination with the professional debridement to prevent recolonization of bacteria and to support patient’s at-home routine. Antiseptics such as chlorhexidine with different formulations and dosages, locally delivered antibiotics (minocycline, doxycycline, lincomycin, erythromycin, and metronidazole) and systemic antibiotics are used [5]. In addition to traditional therapeutic approaches, data on peri-implant mucositis treatment with postbiotic gels and probiotics can be found in the literature [6,7].

At-home oral-hygiene interventions are key for both primary and secondary prevention. The goal of these techniques is to achieve plaque control around implants [5]. In addition to toothbrushes and interdental-cleaning devices, chemical plaque control systems such as 0.12% chlorhexidine mouth rinse, triclosan/copolymer, and sodium fluoride toothpastes may be used as part of the at-home protocols.

Many clinical trials on peri-implant mucositis treatment are available in the literature, and they are based on professional or at-home intervention procedures [5]. The clinical parameters being assessed are bleeding on probing, probing depth, and plaque index, with a mean time of follow-up of 3 to 8 months [5].

A very recent trial on the efficacy of photobiomodulation after non-surgical mechanical debridement in patients with peri-implant mucositis evaluated cortisol levels in peri-implant sulcular fluid, modified plaque index, modified gingival index, and probing depth. Interestingly, over a short-term of follow-up, the use of photobiomodulation did not show additional benefits in terms of reducing soft-tissue inflammatory parameters and cortisol levels [8].

Photobiomodulation is a non-invasive, safe and cost-effective therapeutic option widely described with effects on mucosal and skin tissues: (i) acceleration of healing times; (ii) induction on neovascularization; (iii) pain control; and (iv) reduction in inflammation. In particular, Bioptron Hyperlight Therapy has been shown to be effective in microcirculation improvement, periodontal tissue regeneration, decrease in inflammation, and pain and stress relief, without any side-effects [9]. In fact, its use has been added to traditional clinical protocols of non-surgical periodontal therapy and recent clinical studies on adult chronic periodontitis; mucosal ulcerative lesions and orofacial trauma management showed a significant improvement in clinical parameters and a better patients’ response to treatment [10,11,12]. Moreover, a randomized controlled trial on photobiomodulation as an adjunct to non-surgical mechanical debridement for peri-implantitis showed a significant reduction in modified plaque index (mPI), PD, and bleeding on probing, compared to mechanical debridement alone [13]. On the other hand, a systematic review on photodynamic and laser therapy in peri-implant mucositis treatment showed inconclusive findings, mainly due to the high heterogeneity reported in the studies. In this clinical scenario, due to the scarcity of studies and the mixed results reported on photobiomodulation and peri-implant mucositis, it is not possible to provide evidence-based clinical recommendations.

The null hypothesis of this study is that adding Bioptron Hyperlight Therapy does not provide better clinical outcomes than the standard of care alone.

The primary and secondary aims were to prospectively evaluate in a cohort of patients with a diagnosis of peri-implant mucositis: (1) the efficacy of professional mechanical debridement alone compared with professional mechanical debridement plus the adjunctive use of photo-biomodulation with the use of Bioptron Light therapy and (2) the changes of SAT test values in the treatment and control groups.

## 2. Materials and Methods

The protocol was approved by the Policlinico di Bari, Università degli Studi Aldo Moro Ethical Committee (approval number n.3464/2016 and date 23 December 2016) and informed consent was obtained from all individuals. All the procedures were in accordance with the 1964 Helsinki Declaration and its later amendments or comparable ethical standards. The experimentation followed CONSORT guidelines and was registered at the US National Institutes of Health (ClinicalTrials.gov accessed on 3 March 2022) #NCT05307445.

### 2.1. Study Design

A randomized clinical trial was conducted. The analysis included data from all patients with different stages of peri-implant mucositis. After enrolment, the patients were divided into two groups: Study (SG) and Control (CG).

### 2.2. Study Population and Setting

Enrolment of the subjects was conducted at Policlinico di Bari, Università degli Studi Aldo Moro, Bari, Italy. The enrolled subjects were subsequently divided into two groups: Control Group (*n* = 20, professional mechanical debridement) and Study Group (*n* = 20, professional mechanical debridement + adjunctive use of photo-biomodulation with the use of Bioptron Light therapy). The study was conducted at patient and implant level. An implant was selected for each patient showing clinical signs of mucositis with the highest clinical parameters. An intraoral radiograph was performed to rule out the clinical hypothesis of peri-implantitis. The study covered the same implants, namely Intralock Gold Blue, with a surface impregnated with CaPO_4_, with screw-on prosthesis and selected positions in the oral cavity (second premolar and/or first molar).

### 2.3. Sample Size Calculation

Sample sizes were determined for the probability of the Type I error α = 0.05, the power 1 − β = 0.8. Concerning the variable PPD (primary outcome), the expected difference between the means was supposed to be 0.5 with a standard deviation of 0.5 [14]. Effect size—ratio of mean difference to standard deviation—equal to 1. This gives *n* = 16 in each group [15].

### 2.4. Inclusion Criteria

1. Male or female, 30–60 years old.
2. With diagnosis of peri-implant mucositis.
3. Plaque index (PI) ≥ 40%.
4. Al least one implant site with PPD ≥ 4 mm, BoP+ and suppuration.
5. No uncontrolled diabetes, cardiovascular diseases, bone metabolism disorders, no autoimmune diseases (lichen planus, pemphigoid, pemphigus, and systemic lupus erythematosus).
6. No pharmacological therapies, no chemo-radiotherapies.
7. No smoking (>10 cigarettes/day), alcohol, and/or drug consumption.
8. No pregnancy or breastfeeding.
9. No allergies.

### 2.5. Randomized Allocation

The enrolled patients were randomly assigned to the SG or CG. A randomized allocation was carried out by computer-generated randomly permuted blocks (generated by WH and sealed in envelopes).

### 2.6. Blinding

Blinding of data collection at baseline before allocation and outcome adjudication at all times of follow-up was achieved.

### 2.7. Patient Dataset and Periodontal Charting

The following data were collected: gender, age, Body Mass Index (BMI), caries experience (DMFT), and smoking.

A blinded operator to the allocation procedure and with documented skills in oral hygiene completed the periodontal chart at T_0_ and collected the data at all times of follow-up in order to avoid inter-personal variations. Probing pocket depth (PPD), plaque index (PI), bleeding on probing (BoP), and pain intensity reduction on the visual analogue scale (VAS) were assessed and recorded. VAS scale magnitude estimation requires subjects to indicate the level of pain along a continuum represented by a visual analogue scale (VAS).

### 2.8. Duration of the Study and Times of Follow Up

The study period was 6 months. Patients were treated at baseline (T_0_) and at 6 weeks (T_1_), 12 weeks (T_2_), and 24 weeks (T_3_). Periodontal indices were recorded at all times of follow-up and a database was collected.

### 2.9. Salivary SAT-Test Assessment

For the present study, a photometer was used with an incorporated centrifuge (FRAS 5 EVOLVO: Free Radical Analytical System, H&D SPA, Parma, Italy) to perform the Salivary Antioxidant Test (SAT) for the evaluation of total oxidative stress. A salivary sample was collected from fasting patients after at least 30 min from daily dental hygiene; in fact, foods and beverages or the use of toothpaste can produce an alteration in antioxidant agents in the saliva. Due to the circadian rhythm, the saliva collection was planned in the morning, scheduled from 10.00 to 11.30. The patient was asked to spit into a test tube for 5 min, and then 2 mL of saliva was collected with a calibrated pipette.

The reference values for the SAT test are expressed in mEq/L of antioxidant agents.

<1000 mEq/L: High Deficiency
1000–1500 mEq/L: Optimal Values
1500–2000 mEq/L: Normal Values
2000–2500 mEq/L: Borderline Values
>2500 mEq/L: Possible ongoing inflammation

For the study, a total of 160 salivary samples have been collected; four for each patient at T_0_, T_1_, T_2_, and T_3_.

### 2.10. Description of the Bioptron Light Therapy

Patients in the SG received Polarized Polychromatic Incoherent Low Energy Radiation using a Bioptron^®^ Device (Zepter; Wollerau, Switzerland) [7]. The Bioptron^®^ Device provides polarized visible polychromatic noncoherent light with 90 W; light wavelength = 480–3400 nm; degree of polarization = 95%; specific power = 40 mW/cm^2^; and energy density = 2.4 J/cm^2^. The duration of each treatment session was 10 min. Bioptron^®^ Device was located 10 cm from the oral mucosa and a mouth opener was used. Two weekly sessions during the first 4 weeks of treatment were scheduled.

### 2.11. Professional Mechanical Debridement: Clinical Procedure

At T_0_, T_1_, T_2_, and T_3_, debridement treatment performed with the Mectron Comby Touch piezoelectric scaler (Mectron Combi Touch, Mectron Spa, Carasco (GE), Italy) with a special insert for implants (implant cleaning SET S, Mectron Spa, Carasco (GE), Italy).

Periodontal chart parameters (PI, BoP, and PPD) were assessed using the PCP 15 mm periodontal probe (Hu-Friedy, periodontal probe graduated at intervals of millimeters, bold marks at 5 mm, 10 mm, and 15 mm).

### 2.12. D-BioTECH Approach

All patients, at baseline and all times of follow-up, followed the D-BioTECH approach [12]. This approach allows visualization of the topography of the oral biofilm, with a triton plaque detector (GC TRI PLAQUE ID GEL, GC corporation, Tokyo, Japan) with red/pink colour for a recently formed bacterial biofilm; blue/purple colour for a mature bacterial biofilm over 48 h; and light blue/blue colour for high-risk areas where the bacteria were most active, highlighting their acidic ph. The D-BioTECH approach makes it possible to share the location of the biofilm with the patient and to provide personalized instructions on biofilm removal treatments using dedicated tools. In addition, it allows for an ergonomic use of powders (Powder Sensitive, Mectron Spa, Carasco (GE), Italy) and a reduction in environmental pollution. D-BioTECH approach was performed with the Mectron Comby Touch piezoelectric scaler (Mectron Combi Touch, Mectron Spa, Carasco (GE), Italy) with insert (Insert S1S universal and then insert P3, Mectron Spa, Carasco (GE), Italy).

### 2.13. Co-Intervention

The oral care of the patients was standardized: the same toothpaste (Ialozon toothpaste, Gemavip, Italy), toothbrush (GUM^®^ Sonic Sensitive, medium bristles, Sunstar Europe, Etoy, Switzerland), and interproximal cleaner (GUM Soft-Picks Advanced, Sunstar Europe, Etoy, Switzerland) were delivered to all subjects, toothbrushing indications were 3 times/day for at least 2 min. Moreover, home application of: (a) rinses with mouthwash based on ozonated extra virgin olive oil (Ialozon Blu, Gemavip, Italy), three applications a day for a week (12 mL, rinses of 30 s), then morning and evening for the entire treatment period and (b) an intra-oral gel based on ozonated extra virgin olive oil (Ialozon Oral Gel, Gemavip, Italy), 3 times a day for 7 days, and then in the morning and evening for the entire treatment period. Patients received instructions for the at-home oral hygiene procedures based on the Tailored Brushing Method (TBM) [16,17,18].

### 2.14. Statistical Analysis

Welch’s two sample *t*-tests were used to test the difference in changes of numerical characteristics in consequent time intervals and during all the time of observation [T_0_; T_3_]. Pearson’s Chi-squared test of homogeneity was used to test the difference in BoP in CG and SG. Shapiro–Wilk normality test was used to assess normality of data distribution. The results were considered statistically significant at *p* < 0.05. The R statistical program, ver.4.1.2 (The R Foundation for Statistical Computing, Wirtschaftsuniversität Wien, Vienna, Austria) was used for the statistical analyses.

## 3. Results

### 3.1. Population Characteristics

**Figure 1:**
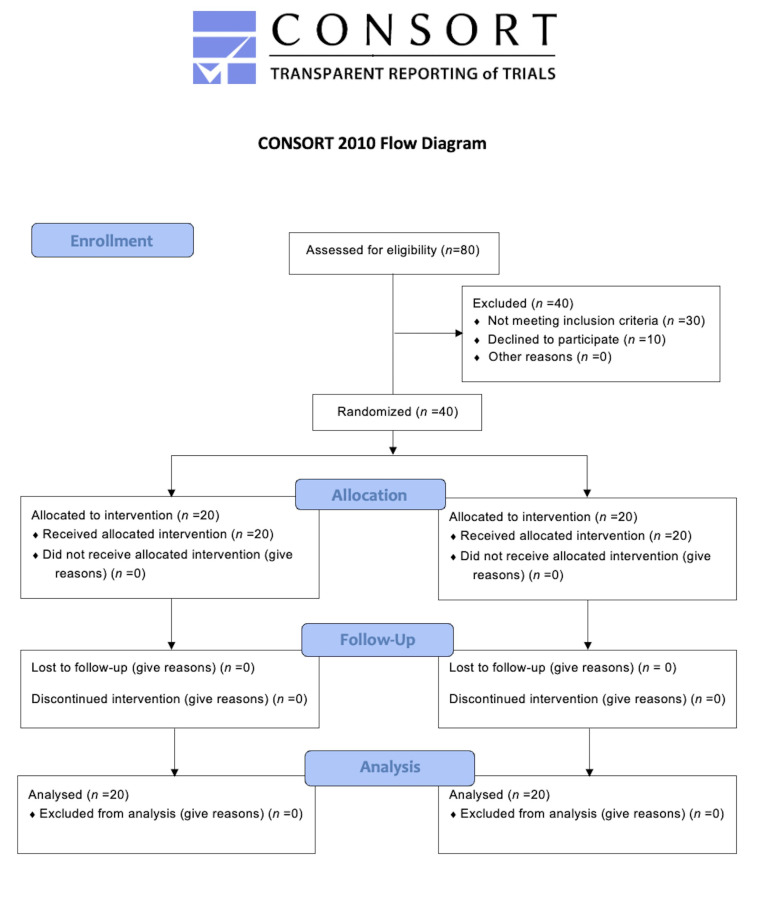
Flow chart of the study.

Eighty patients were evaluated at the enrolment stage, then 40 subjects met the inclusion criteria, and they were randomly assigned to SG and CG (20 patients in each group). Figure 1 shows the flow chart of the study. PI, PPD, BoP, and SAT were measured for each patient at T_0_ (baseline), T_1_ (6 weeks), T_2_ (12 weeks) and T_3_ (24 weeks). Distribution parameters of numerical and categorical characteristics in the groups are shown in Table 1 and Table 2 accordingly.

### 3.2. Plaque Index

**Figure 2:**
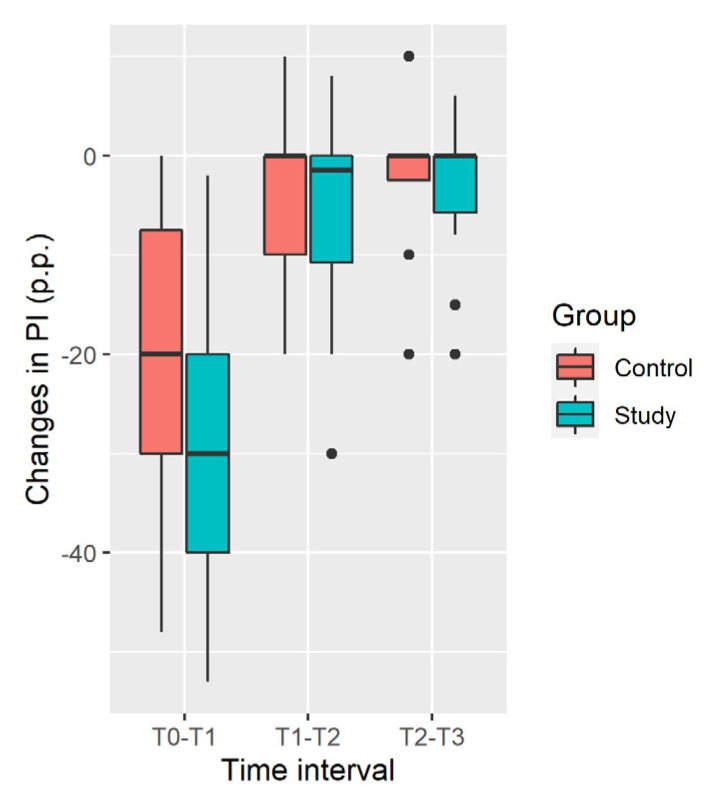
Distribution of changes in plaque index (percentage points).

Distribution of changes in the plaque index in the Study and Control groups are shown in Figure 2.

Plaque Index decrease in the Study Group is significantly larger at the [T_0_; T_1_] time interval and during all the time of observation [T_0_; T_3_], while insignificantly larger in the [T_1_; T_2_] and [T_2_; T_3_] intervals (Table 3).

### 3.3. Probing Pocket Depth

**Figure 3:**
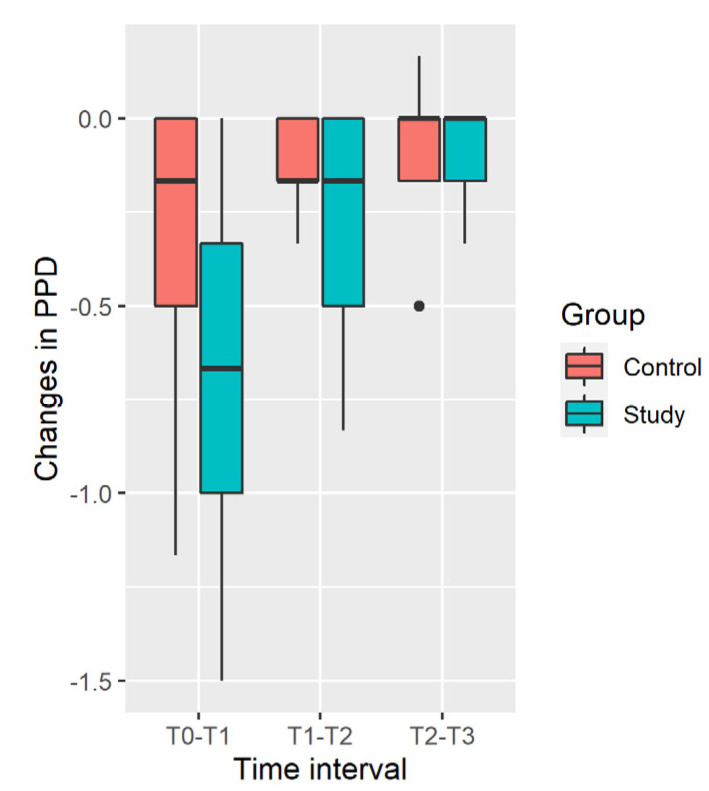
Distribution of changes in PPD.

Distribution of changes in the PPD in the Study and Control groups is shown in Figure 3.

PPD decrease in the Study Group is significantly larger in the [T_0_; T_1_] and [T_1_; T_2_] time intervals and during all the time of observation [T_0_; T_3_], while difference is insignificant in the [T_2_; T_3_] interval (Table 4). Results should be taken with caution, due to significant deviation from normality of distribution in the Control Group (*p* = 0.04 in the [T_0_; T_1_] time interval, *p* = 0.01 in the [T_0_; T_3_] time interval).

### 3.4. Salivary Sat Test

**Figure 4:**
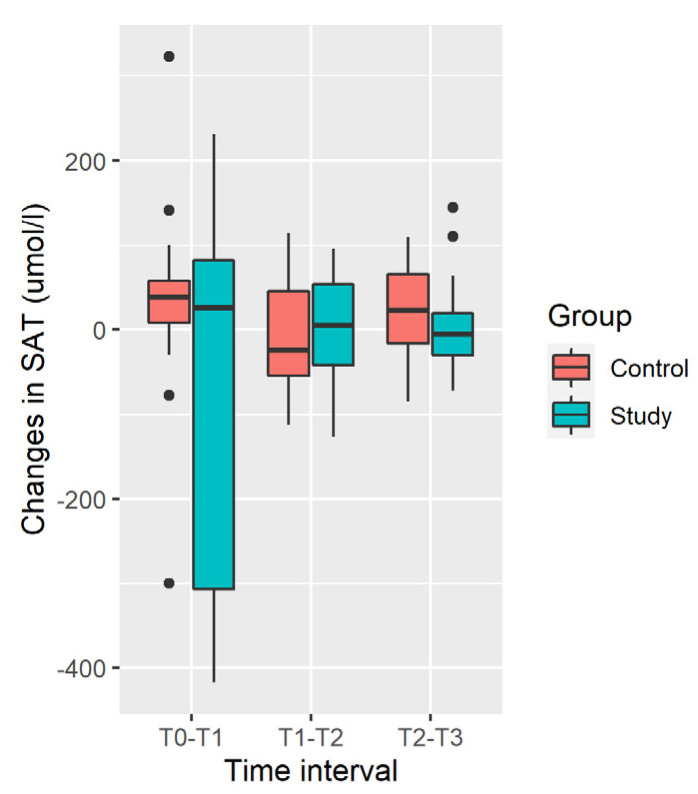
Distribution of changes in SAT.

Distribution of changes in the SAT in the Study and Control Groups is shown in Figure 4.

Increase in mean SAT was observed in the Control Group, and decrease was observed in the Study Group. Differences in SAT changes are not significant at any time interval and during all the time of observation [T_0_; T_3_] due to the large variability of changes (large SD, Table 5).

### 3.5. BoP

As shown in Table 2, BoP cases decreased faster in the Study Group than in the Control Group. There were no cases of BoP in the Study Group already at T_2_, while there were 8 out of 20 cases in the Control Group even at T_3_. Pearson’s Chi-squared test of homogeneity confirmed that this difference is significant (*p* = 0.004).

### 3.6. Pain Intensity Reduction

Comparing the VAS score at T_1_ and T_2_ in the two groups, a significant improvement was recorded in patients undergoing Bioptron Hyperlight Therapy adjunctive treatment (T_1_  =  2.9 vs. 5.5; *p*  <  0.05 and T_2_  =  1.5 vs. 3.2; *p*  <  0.05). The baseline VAS score in the SG and in the CG was 6.6 and 7.1, respectively.

### 3.7. Influence of Patients’ Characteristics

Influence of patients’ gender and smoking on changes in PI, PPD, and SAT were checked by Welsh two sample *t*-tests. No significant difference was detected. Influence of patient’s age, BMI, and DMFT on changes in PI, PPD, SAT were checked by Pearson’s correlation test. Patient’s age is significantly negative correlated with SAT in the Study Group. This means that elder patients commonly have a larger decrease (or smaller increase) in SAT. The rest of the correlations were insignificant.

## 4. Discussion

This clinical trial assessed the efficacy of the adjunctive use of Bioptron Hyperlight Therapy in peri-implant mucositis treatment compared to mechanical debridement alone.

Periodontal indexes (PPD, PI, and BoP) decreased significantly in the Study Group compared with the Control Group. SAT-test variables increased in the CG and decreased in the SG, but the changes were not significant at any time of follow-up. Among the patients’ dataset (age, gender, DMFT, BMI, and smoking), only the age factor significantly correlated with SAT in the Study Group, with evidence that elder patients commonly have a larger decrease (or smaller increase) in SAT values.

Peri-implant mucositis is a biofilm-induced inflammatory condition around dental implants. It is characterized by local swelling, redness, soft tissue surface shininess, and suppuration, combined with any bleeding on probing and an increase in probing depths. Moreover, the patient may complain soreness. Intra-oral radiographic evaluation does not show evidence of bone loss beyond those changes resulting from the initial alveolar bone remodelling process after implant placement [4].

The standard of care treatment of peri-implant mucositis is based on the mechanical removal of biofilm around implants and prosthetic surfaces, through professional and at-home interventions, with possible additional use of antimicrobial agents [5].

A cause-and-effect relationship between the experimental accumulation of biofilm and the development of peri-implant mucositis in humans was demonstrated [19]. Peri-implant mucositis is a reversible condition related to environmental equilibrium. Experimental studies showed that the biofilm accumulation for a period longer than 3 weeks causes: (i) inflammatory migration of leukocytes through the epithelium barrier and (ii) the creation of an infiltrate with an enlarged proportion of T- and B-cells in the connective tissue next to epithelium barrier [20,21]. The onset of peri-implant mucositis can be described by both histology and immunohistochemistry, and tissue inflammation follows expression of inflammatory markers [22,23].

The process of biofilm formation and bacterial colonization on implant surfaces is a complex physicochemical phenomenon. Understanding of oral health and disease by oral microbiota alterations offers a holistic viewpoint that is less focused on single or a few pathogens [24,25]. Peri-implant mucositis is associated with an increased presence of cocci, spirochetes, and motile bacilli and it may result in an increase in the proportion of periodontopathogen bacteria, mostly from the orange complex: *F. nucleatum*, *P. intermedia*, and *Eubacterium* species and a decrease in *Streptococci* spp. and *Actinomyces* spp. [26,27,28]. Peri-implant mucositis is a nonspecific, polymicrobial, and heterogeneous disease with an endogenous etiology. Despite the fact that it is a potentially reversible condition, it is also a transitional event in the progression to peri-implantitis [29]. Therefore, attention should be paid to identify this condition at the earliest stage.

Periodontal indices are used for peri-implant mucositis stage, but in complex clinical situations, they may not be sufficient to describe the condition clinically. More sensitive and repeatable diagnostic protocols involving molecular, microbiological, clinical, and radiological tools are required. For example, specific biomarkers have been recognized and can be useful in early diagnosis of the disease (e.g., increase in apolipoprotein-100, IL-8), monitoring the infection over time (e.g., increased level of pro-inflammatory cytokines), or its progression (e.g., increased level of cytokines followed by the markers of bone resorption and specific “red complex” markers), as well as the resolution phase (e.g., decrease in pro-inflammatory cytokines and ICTP, increase in neutrophil defensins). The improvement of diagnostic protocols, a better understanding of the epidemiology, risk management, and personalized patient care are still needed [30,31,32].

In this clinical scenario, we proposed a clinical protocol for the treatment of peri-implant mucositis with the aid of Bioptron Hyperlight Therapy. The results showed that application of Bioptron resulted in a faster improvement of all the inflammatory parameters at all the times of follow-up.

Bioptron is widely used in Central and Eastern Europe, and it was firstly proposed by Hungarian scientist Mester. It is a non-invasive, safe, and cost-effective therapeutic option for the treatment of wounds, pain of musculoskeletal system, or dermatological disorders [10,33,34,35,36]. Bioptron light therapy system is a device with an optical unit emitting light that is similar to the sun, in terms of the electromagnetic spectrum produced, but without UV radiation. Bioptron irradiation increases the metabolism in human blood cells, affecting their cytokine production and driving the immune response towards an anti-inflammatory/reparative profile in addition to mitochondria activation and pain reduction [10,34,37]. These factors support the regenerative processes of irradiated tissues. However, a significant drawback of this procedure is the frequency of obligatory visits and the relatively long cumulative exposure time needed to obtain clear results. This requires significant involvement, above all, on the part of the patient. The model used in this case report is a B2 device which is relatively heavy (0.5 kg) [10,38] making it difficult to apply the procedure, which usually lasts several minutes or longer.

The results of clinical studies in the literature are contradictory and it is not currently possible to provide evidence-based clinical recommendations for treatment of peri-implant mucositis with the use of photobiomodulation [6]. In fact, a systematic review reporting the evidence on photodynamic and laser therapy in the management of peri-implant mucositis showed that the heterogeneity of the included studies was high due to methodology bias and that more robust clinical trials are needed [14].

In this scenario, this study adds new elements on the efficacy of Bioptron therapy in adult patients with peri-implant mucositis. Compared to other photobiomodulation technologies, Bioptron is a tool “independent of the operator”, and it does not require procedures to be performed by the clinician, but simply must be directed, set up and activated for the mandatory time and number of sessions. It can therefore be defined as an ergonomic, non-invasive clinical procedure that is highly appreciated by patients for the relief reported in oral tissue inflammation [38].

### Limitations

This clinical study is based on a limited number of patients and without any further classification of the mucositis stage. Further research is needed to verify if the implant system may be itself a prognostic factor in the aetiology and treatment of peri-implant mucositis. In addition, personalized medicine with patient groups classified by age and gender, to take into account subjective characteristics such as the endocrine system, aging, and lifestyle, is awaited.

## 5. Conclusions

Based on the findings of this clinical trial, the adjunctive use of Bioptron light therapy is successful in the treatment of peri-implant mucositis compared to mechanical debridement alone. Bioptron light therapy was shown to be effective in the reduction in the patients’ inflammatory indicators, pain expressed on VAS, and in the healing of soft tissue around implants. Future work on using photobiomodulation to treat peri-implant mucositis will need to focus on a personalized approach. To do this, it is necessary to develop large clinical trials with very precise parameters to identify risk and prognostic factors for each type of patient.

## Figures and Tables

**Table 1:** Numerical characteristics of Study Group and Control Group.

	Study Group				Control Group			
	Min	Max	Mean	SD	Min	Max	Mean	SD
Age (yrs)	36	74	56.7	9.131	29	76	51.2	10.94
BMI (kg/m^2^)	17.55	34.43	25.62	4.777	20.75	35	27.17	4.303
DMFT	9	24	17.35	5.071	5	24	14.2	5.845
Plaque Index								
T_0_	40%	90%	64%	12%	20%	76%	52%	16%
T_1_	10%	60%	34%	14%	15%	50%	33%	9%
T_2_	10%	50%	27%	10%	20%	60%	31%	9%
T_3_	10%	38%	23%	9%	20%	40%	29%	6%
PPD (mm)								
T_0_	3	7	4.317	1.101	2.667	5.167	3.858	0.674
T_1_	2	6.833	3.633	1.148	2.667	5	3.542	0.642
T_2_	2	6	3.375	1.079	2.5	5	3.433	0.685
T_3_	2	6	3.275	1.041	2.333	5	3.375	0.658
SAT test (mEq/L)								
T_0_	254	1325	674.7	330.1	271	1217	663.3	248.2
T_1_	326	1120	592.8	221.7	320	1235	694.2	238.2
T_2_	375	993	598.5	187.8	342	1188	680.5	211.8
T_3_	423	997	604.5	168.1	312	1201	705.7	202.7

**Table 2:** Categorical characteristics of Study Group and Control Group.

	Study Group		Control Group	
Gender	F: 8	M: 12	F: 9	M: 11
Smoking	No: 11	Yes: 9	No: 13	Yes: 7
**BoP**				
T_0_	20		20	
T_1_	4		8	
T_2_	0		9	
T_3_	0		8	

**Table 3:** Comparison of Plaque Index changes. Mean and SD in percentage points.

Time Interval	Study Group		Control Group		*p*	95% CI
	Mean	SD	Mean	SD		
[T_0_; T_1_]	−29.85	11.55	−19.05	15.48	0.017 *	(−19.56, −2.036)
[T_1_; T_2_]	−6.60	10.92	−2.75	7.525	0.203	(−9.877, 2.177)
[T_2_; T_3_]	−4.25	7.926	−2.00	6.959	0.346	(−7.027, 2.527)
[T_0_; T_3_]	−40.70	13.03	−23.80	15.88	0.001 *	(−26.21, −7.587)

*—significant difference.

**Table 4:** Comparison of PPD changes. Mean and SD of average value of six measurements in each tooth.

Time Interval	Study Group		Control Group		*p*	95% CI
	Mean	SD	Mean	SD		
[T_0_; T_1_]	−0.683	0.415	−0.317	0.390	0.006 *	(−0.624, −0.109)
[T_1_; T_2_]	−0.258	0.278	−0.108	0.098	0.032 *	(−0.286, −0.014)
[T_2_; T_3_]	−0.100	0.137	−0.058	0.189	0.431	(−0.148, 0.064)
[T_0_; T_3_]	−1.042	0.393	−0.483	0.389	<0.001 *	(−0.809, −0.308)

*—significant difference.

**Table 5:** Comparison of SAT changes. Mean and SD in mEq/L.

Time Interval	Study Group		Control Group		*p*	95% CI
	Mean	SD	Mean	SD		
[T_0_; T_1_]	−81.9	221.1	30.95	111.5	0.051	(−226.3, 0.5644)
[T_1_; T_2_]	5.75	61.24	−13.70	64.96	0.336	(−20.97, 59.87)
[T_2_; T_3_]	5.95	53.98	25.15	54.36	0.269	(−53.88, 15.48)
[T_0_; T_3_]	−70.2	259.2	42.4	132.6	0.095	(−245.9, 20.67)

## Data Availability

Study data are available. They must be officially required from the corresponding author.
